# Alternative Perspectives: How Chinese Medicine Understands Hypercholesterolemia

**DOI:** 10.1155/2010/723289

**Published:** 2010-08-11

**Authors:** Kylie A. O'Brien

**Affiliations:** ^1^Department of Medicine, Monash University, Prahran, VIC 3800, Australia; ^2^Faculty of Health, Engineering & Science, Victoria University, P.O. Box 14428, Melbourne, St Albans, VIC 8001, Australia

## Abstract

Treatment of cardiovascular disease, albeit under the auspices of other clinical descriptors to those described in western biomedicine, has a long history in China. Chinese Medicine (CM) is guided by unique philosophical underpinnings and theories. There are differences in how the heart is conceptualised traditionally in CM compared to biomedicine. This paper focusses on how hypercholesterolemia is understood from within the Chinese medical paradigm, including its aetiology, pathogenesis, and treatment. A brief overview of the key characteristics and theories of CM is given to provide context. Modern science has demonstrated that many Chinese herbs have cholesterol-lowering properties. Examples of research into individual herbs and medicinal formulae, combinations of herbs are presented. At a more sophisticated level, some researchers are challenging some of the very assumptions upon which CM is based, including applicability of CM theory to modern clinical entities such as hypercholesterolemia, and are seeking intersections of knowledge between CM and biomedicine that may extend CM theory.

## 1. Introduction

Cardiovascular disease (CVD) has been treated for hundreds, if not thousands of years with Chinese medicine (CM), under the auspices of different clinical descriptors to those used in modern biomedicine. For example, in ancient China the term “xiao ke” described “thirsting and wasting disorder”, what is now known as diabetes. Whilst western biomedicine coexists with CM within China, it remains that within the field of CM there is a unique understanding of the heart and cardiovascular system that influences how CVD is treated with Chinese herbal medicine and acupuncture. As new clinical entities have emerged in modern times, and as CM has sought to integrate with western medicine, CM theories have been applied to understand and justify treatment of such diseases or disorders. Hypercholesterolemia is one such example—it is a biomedically defined clinical entity, a risk factor for CVD with no historical precedence of treatment hundreds of years ago. 

This paper discusses how hypercholesterolemia is understood within CM, including aetiology, pathogenesis, and treatment. It is necessary for the reader to have some understanding of the theoretical framework of CM, therefore I will begin with a brief summary of the main guiding theories and key characteristics of CM, along with a description of the components of the cardiovascular system. There is increasing scientific evidence that many Chinese herbs lower cholesterol and have other positive effects on the cardiovascular system; information about individual herbs and medicinal formula is included. Finally, there is a discussion about some creative research that attempts to investigate potential intersections of CM and western medicine, along with the merits of such investigations. 

It is important that the reader keep an open mind—whilst some of the CM terminology may appear unsophisticated and naive, in some ways this apparent simplicity masks a profound complexity that takes years to understand deeply and master. Unlike western medical language, much of CM terminology was derived from everyday language. I have, through necessity, simplified descriptions—this paper has been written for western medical colleagues with a more limited knowledge of CM. Note that the concept of an organ differs from the biomedical one, and so organ names are capitalised when referred to within the context of CM.

## 2. Chinese Medicine Features and Guiding Theories

### 2.1. Features of Chinese Medicine

There are some important distinguishing features of CM that set it aside, in particular, from western medicine. It is underpinned by a worldview that is materialistic and dialectic and an ideology of holism. The notion of interdependence permeates the understanding of the functioning of the organ systems within the body in health and illness and describes the relationship between humans and the environment. Flowing from the holistic philosophy is the understanding of health as a reflection of harmony: between humans and nature, between the organ systems, tissues, and vital substances of the body, and the mind and emotions [1]. Consideration is given to the root cause (the “ben”) and the symptoms (the “branch” or “biao”) of an illness, with treatment aiming to address the root cause, not just the symptoms. Treatment also is individualised to the patient, taking into account such factors as constitution, age, gender, and season of occurrence of the illness.

Another characteristic of the medicine has been to differentiate diseases (or signs or symptoms) according to underlying “patterns of disharmony”, termed syndromes. Each syndrome is characterised by particular signs and symptoms, reflective of the underlying pathogenesis at that point in time. Treatment aims to treat both disease and underlying syndromes. Chinese herbal medicines (CHMs), typically combinations of 4–12 different herbs, are usually syndrome-specific. A CHM designed to treat one syndrome will usually contain very different herbs to a CHM designed for another syndrome (of the same disease). Although this syndrome differentiation has become thought of as a central feature of CM during the last century, prior to this time there are many recorded examples where reference is made to simply treating the disease [2]. 

Chinese medicine diagnosis is based on collection of clinical data and analysis of signs and symptoms according to several CM theories to arrive at a diagnosis of the disease/disorder and its underlying CM syndrome. The four main diagnostic methods are: taking a case history, inspection (face, hair, body, gait, tongue body, and coating), auscultation (e.g., listening to the voice, breathing sounds, quality of a cough), and palpation (of body parts if indicated, and in particular, pulse diagnosis). Pulse diagnosis involves palpation of the radial artery of both wrists, to ascertain the state of the vital substances of the body, the qi and blood, as well as the various organ systems. 

What I believe is also a distinguishing feature of CM when juxtaposed to western medicine is the co-existence of many and in some cases, seemingly contradictory, theories: there is no imperative for one to be correct or supersede another, perhaps reflective of the differences in underpinning metaphysics of the two systems of medicine. 

### 2.2. Basic Theories

Yin Yang Theory posits that there are two complementary yet opposite forces, termed Yin and Yang, that form the basis of life. Health relies on a harmonious balance of Yin and Yang and illness is seen as an imbalance of these two vital forces. It was believed that Yin Yang Theory originated with peasants' observations of the change in shadow and light on the two sides of a mountain with the apparent passage of the sun in the sky [3]. The concept of Yin became synonymous with cold, rest, and night, and “Yang” was used to describe heat, activity, and day [3, 4]. By way of analogy it was incorporated in CM to describe the body. For example, the exterior of the body was Yang in relation to the interior which was Yin, the upper portion of the body Yang in relation to the lower (Yin). It was also used to describe the normal physiological functioning of the body as well as pathological processes. Signs and symptoms could be categorised as Yang (e.g., a loud barking cough with profuse sputum) or Yin (in comparison, a weak cough with little sputum expectorated). These concepts of Yin and Yang are applied to the organ systems of the body. For example, there is the notion of “Kidney Yin” and “Kidney Yang” denoting different aspects of the Kidney, as it is understood within CM theory.

The notion of “qi” is central to CM: qi forms the basis of all that is, including the human being and the environment. Qi is both a material concept and a functional one. Materially it can be thought of as a kind of subtle, rarefied “energy” of essential life force that permeates the body and environment, forming the material basis of the body. In a functional sense it describes the activities of the “zang-fu” organ systems within the body (discussed later). Though it is more complex than this there are over 270 different definitions of qi in one of the oldest guiding CM textbooks, the *Huang Di Nei Jing* [5]. Qi is believed to circulate within special conduits in the body termed meridians and collaterals that connect all parts of the body, including connecting the organ systems with each other and their related sense organs. Along these meridians lie special points termed “acupoints”, where the qi of the meridians is particularly concentrated [6]. It is through stimulation of these acupoints with needles that acupuncture has its effect. There are several types of qi that perform different functions, and each “zang” organ has its own qi also. 

Meridian Theory is the key theory to describe the distribution of the meridians, their functioning in a physiological sense, and the interrelationships between the meridians and the organs they connect to. There are 12 main meridians in the body, each connecting with one of the major “zang-fu organs” in the body (and named after it) (see later). Qi circulates through the meridians in a defined sequence (beginning with the Lung meridian and ending in the Liver Meridian), and each meridian is at a peak energetically during a specific two-hour period. Meridian Theory provides the basis for understanding both the physiological and pathological functioning (in illness) of the human [7].

Five-Phase (or Five-Element) Theory posited that there were five elements within nature that are fundamental to life: wind, metal, fire, earth, and water, that were taken to symbolise behaviour of phenomena in nature. By observing the elements in nature, analogies were drawn with respect to the functioning of the body in health and disease. The theory was used to categorise phenomena including the seasons, colours, tastes, body organs, sense organs, and emotions. For example, the element “wood” was related to the season of spring, the Liver organ, the eye (sense organ), the tendons and nails, the sour taste, the colour green, and the emotion of anger. Through analysis of signs and symptoms, it is possible to identify involvement of particular elements (and therefore organ systems). The five elements and their associated organ systems exist in specific relationship to each other. These relationships provided a model of understanding how disharmony in one organ system affect others. Through an understanding of these inter-relationships, the practitioner treats not only the element/organ involved in pathology, but often those organs related via these relationships. 

Zang Fu Theory is the major theory to describe the organ systems within the body, termed “zang-fu” organs. The zang organs are the “solid” organs: the Heart, Lung, Kidneys, Liver, and Spleen. Each zang organ is connected to a “hollow” fu organ via the meridian system, for example the Liver with the Gallbladder and the Kidney with the Bladder. Each zang organ is better thought of as a functional organ system, with some functions very different to those in biomedicine, though they do encompass these in modern practice. For example, each zang organ is also connected to a sense organ, and its condition is reflected in an external part of the body. The Liver is related to the eye (and sense of sight) via the meridian connections, and the condition of the Liver is reflected in the nails. Thus, it is often the case in conditions of the eye that the Liver is treated in CM. The function of the Liver is to ensure the “free flow” of qi around the body and to “store blood”. It plays an important role in harmonising the emotions via this ability to ensure qi flow is smooth. 

Yin Yang Theory becomes combined with Zang Fu Theory in descriptions of pathology of the zang-fu organs. For example, there is a Yin and a Yang aspect of the Kidney. The Kidney Yin and/or the Kidney Yang can become deficient (inadequate)—in such cases, the syndrome would be termed “Kidney Yin deficiency” or “Kidney Yang deficiency” respectively. 

With respect to the understanding of aetiology of disease, CM recognises external causes, internal (particularly relating to the emotions) causes, and those that are noninternal and nonexternal (e.g., trauma). There are six climatic factors in nature: wind, fire, summer-heat, dryness, cold, and dampness, that if occurring out of season or with particular force, and the person is susceptible, can become aetiological factors in disease. By way of analogy, particular symptoms and signs may be seen as evidence of the presence of such aetiological factors internally. Internal dampness, for example, can manifest as discharges (e.g., sputum) or oedema. Each organ system has a related emotion which, if excessive or prolonged, can cause problems in its related organ, for example, anger and the Liver. Conversely pathology in a particular organ may manifest as displays of the particular related emotion. Emotions are seen as important aetiological factors in disease. 

## 3. Components of the Cardiovascular System in Chinese Medicine

In order to understand how hypercholesterolemia is conceptualised in CM, it is necessary to understand the components of the cardiovascular system, in particular the Heart and its relationships to other organs. The Heart (“Xin” in Chinese) is the central zang-fu organ involved in the cardiovascular system, with the other zang-fu organs assuming roles due to the interdependent relationships between them. According to Meridian Theory, a number of the meridians and collaterals pass through the Heart including the Heart meridian, Small Intestine meridian (the paired fu organ of the Heart), Kidney meridian, and Spleen meridian [7]. As a consequence of the interdependent relationships, dysfunction of the Heart may affect other zang-fu organs and vice versa. The Heart is said to be the “commander of blood” and “dominates the blood and the vessels,” aphorisms identifying the Heart as the major organ responsible for blood circulation. *Heart qi* provides the motive force for the heartbeat [6]. 

The Heart has a much broader role than blood circulation. The Heart is understood to “*house the shen*” or “house the mind”. Shen, variously translated as “spirit” or “vitality”, refers broadly to the exterior manifestation of physiological functioning of the body and in a narrower sense, to consciousness and mental functioning, including memory, perception, and thinking [3, 6]. A quote from the ancient Chinese text, the *Huang Di Nei Jing Su Wen *(The Yellow Emperor's Classic of Internal Medicine: Basic Questions) illustrates this:

“The heart is the sovereign of all organs and represents the consciousness of one's being. It is responsible for intelligence, wisdom, and spiritual transformation [8].”

Conditions such as insomnia and poor memory are often seen to relate to the Heart and its ability to “house the shen”; a notion that is not really consistent with how insomnia is understood in western medicine. Due to the broader understanding of the function of the Heart in CM, if there is a problem with the Heart, this does not automatically equate with a cardiac disorder though of course it can encompass this. Nonetheless, certain clinical disorders in ancient CM literature were clearly describing CVDs as we understand them in biomedicine. However the signs and symptoms described under that clinical descriptor may not have been exclusive to heart disease. For example, the disorder of “Chest Bi Syndrome (here the word “Syndrome” is used in a manner analogous to how it is used in western medicine, rather than as it is typically used in Chinese medicine: to denote an underlying pattern of disharmony)” (“Jue Xin Pain”), described in the ancient text the *Huang Di Nei Jing* [9], describes the condition of pain in the chest but several biomedical clinical entities may fall under this umbrella such as angina, myocardial infarction, gastritis, esophagitis, globus hystericus. 

The Heart “rules” emotional activities as well as mental activities. The particular emotion related to the Heart is joy; thus, if there is over-excitement that is sudden or prolonged, this can be damaging to the Heart. The state of the Heart is not only reflected in the pulse, but also in the complexion. If the face is very pale, this may be a sign of “Heart- blood deficiency” (though a person may or may not be diagnosed as anaemic). Since the Heart connects to the tongue via the internal meridians, incoherent speech may be seen as a sign of pathology of the Heart [10]. 

It can be argued that the four other “zang” organs (Lung, Kidney, Liver, and Spleen) constitute the cardiovascular system due to their various related functions and their relationships with the Heart. Due to the close relationship between qi and blood (blood carries qi, qi is a component of blood), the Lung, known as the “commander of qi” and responsible for qi circulation, is also involved in promotion of blood circulation [6]. The Liver is involved by virtue of its role in regulating or ensuring the free flow of qi around the body and its role in regulating blood volume (“storing blood”). There is some consistency between this understanding of organ connections in CM and that within biomedicine: the connection between the heart and the liver organ in biomedicine is illustrated in portal hypertension, for example. According to CM, one of the Kidney's roles is in fluid metabolism. According to theory, the Kidney and Heart share a special relationship, keeping each other “in check” [6]. The link between Heart and Kidney in CM is shared in biomedicine with the recognition of the renal-aldosterone system in hypertension and heart disease [10]. The Spleen generates “nutrient qi” that circulates through the meridian system and constitutes blood. A function of the Spleen in CM is to “hold” blood within the vessels; if the Spleen is operating suboptimally, bleeding disorders may occur [6]. 

Thus is can be seen that although the Heart is the major organ in the cardiovascular system within CM, the other major “zang” organs are involved by way of their various relationships with the Heart.

## 4. Hypercholesterolemia in Chinese Medicine

### 4.1. Aetiology and Pathogenesis

There is no ancient precedence in CM for treatment of hypercholesterolemia; it is a relatively modern, biomedically defined clinical entity. The understanding of aetiology and pathogenesis of hypercholesterolemia has not been built up over hundreds of years of empirical observation, unlike other diseases or disorders. Explanations of aetiology and pathogenesis according to CM theory have developed relatively recently. Nonetheless, syndrome differentiation has been applied, and various authors have reported typically 3–8 different syndromes of hypercholesterolemia [11–14]. As mentioned previously, treatment with CHM or acupuncture is typically aimed at the disease/disorder and the CM syndrome. 

The pathogenesis of hypercholesterolemia is believed to involve the production of internal “dampness” and “phlegm” that leads to internal obstruction including “qi stagnation” and “blood stagnation”. The three main zang organs involved are believed to be the Spleen, Liver, and Kidney [11] though the Heart is obviously involved by virtue of the fact that it controls the circulatory system. The fundamental pathogenesis of hypercholesterolemia according to Fu [14] is both Spleen and Kidney deficiency and “phlegm” and “blood stagnation.” Spleen qi deficiency is believed to be the most important factor in pathogenesis of hypercholesterolemia, consistent with theory which holds that “dampness” and “phlegm” are produced by a malfunctioning Spleen [14]. The mechanisms by which the aetiological factors create the pathogenesis of the disorder are complex. These factors are believed to include poor diet that leads to malfunctioning of the Spleen and production of internal dampness and phlegm. Emotional upsets, in particular anger, may lead to Liver qi stagnation [12]. Qi stagnation can lead to blood stagnation. Kidney and Spleen Yang deficiency can also lead to internal dampness [12]. Liver and Kidney Yin deficiency may also be involved [11]. 

In CM, phlegm (Chinese “tan”) is considered a secondary pathogenic factor that is both a product and cause of disease created by a dysfunction of one or more of the zang organs involved in fluid metabolism in the body. These organs include the Spleen, the Kidney, and the Lungs. Ancient texts described phlegm as a process of condensing and congealing [15]. More modern texts distinguish between tangible and intangible forms of phlegm. An example of tangible phlegm is nasal discharge whilst an example of intangible phlegm is (the manifestation of) dizziness. The concept of phlegm is related to “dampness”; phlegm originates from it [15]. Phlegm creates problems in the body by stagnating or blocking. For example, if phlegm blocks the Lungs, asthmatic breathing may result. If it blocks the meridians, stroke may result [6]. It is not difficult to see the potential relationship between an atheromatous plaque on a blood vessel wall and serum cholesterol and the concept of phlegm in CM. 

Blood stasis (Chinese “yu xue”) refers to a retardation of blood circulation, locally or systemically. Here's where the pathogenesis gets interesting. Qi and blood stagnation may occur as a result of phlegm “blocking.” Qi stagnation may also be part of the pathogenesis of phlegm. Qi stagnation may lead to blood stagnation and vice versa (blood carries qi); qi and blood stagnation often occur concurrently. A deficiency of qi in the body may lead to a deficiency of blood; qi provides the motive force for blood circulation, so if it is deficient, blood circulation may become retarded. Pathogenic cold (i.e., external and enters the body or is internally generated) may block the flow of qi and blood, leading to blood stasis. Pathogenic heat may “congeal” the blood, leading to blood (and qi) stasis. Key organs involved are the Heart (“ruler of blood”) and the Liver (“stores the blood”, involved in circulating qi around the body freely). Thus, there are many pathomechanisms by which blood stasis may occur according to CM. Signs and symptoms that are characteristic of “blood stagnation” include a purple tongue body or petechiae on the tongue, a particular type of pulse quality, purplish lips, a dark complexion and, in the case of pain, a stabbing quality in a fixed location [6].

Through analysis of signs and symptoms, the CM practitioner diagnoses the underlying pattern(s) of disharmony. Herbs are then chosen to make up the medicinal formula accordingly. Chinese herbs are categorised according to their functions and effects on the body (i.e., in turn determined by the properties, nature, and temperature characteristics of the herbs). There are several categories of herbs that address or resolve dampness and phlegm in the CM Materia Medica. In keeping with CM theory of treating the root cause, other herbs will be chosen to address the root cause of the phlegm, for example, Spleen qi deficiency. There are also herbs that have a specific function of addressing “qi stagnation” whilst others address “blood stasis” (those that promote qi and blood circulation). Again, herbs would be added to address the root cause of the blood stasis. If the blood stasis was due to “internal cold,” herbs of a warm nature that “warm the interior” may be added (and herbs overly cold in terms of temperature characteristic avoided, in general). 

### 4.2. Underlying Patterns of Disharmony

Attempts have been made to apply the concept of pattern differentiation (of syndromes) to hypercholesterolemia (bian zhen lun zhi). Various texts have listed between three and eight subcategories (CM syndromes) of hypercholesterolemia [11–13], including “Liver Qi Stagnation and Spleen Deficiency pattern,” “Internal Dampness-Heat pattern,” and “Spleen and Kidney Yang Deficiency pattern” [11–13]. One Chinese study attempted to empirically ascertain the CM syndromes of hypercholesterolemia, however unfortunately the study population contained patients with a myriad of concomitant cardiovascular disorders, rendering it impossible to make any conclusions regarding syndromes of hypercholesterolemia [14]. An interrater reliability study of CM diagnosis in 45 hypercholesterolemic Australians who were otherwise healthy did not find 3–8 neat CM syndromes, instead identifying over 15 different syndromes over the study population [16]. Little agreement on syndrome diagnosis according to Zang-Fu Theory was found between three CM practitioners [16]. These findings lead the researchers to question the applicability of syndrome differentiation to hypercholesterolemia; the syndromes identified may have simply reflected the state of that patient's body at the time or their constitutional pattern and not reflected the pathogenesis relating to hypercholesterolemia [16]. 

It is not yet established with certainty that the concept of differentiation of CM syndromes is in fact applicable to a condition such as hypercholesterolemia. It is possible that hypercholesterolemia is characterised by a complex mixture of syndromes. It is also possible that the underlying pathomechanism of hypercholesterolemia is similar for all people, that is, it is not syndrome-specific [16]. More research is needed. Why this is important is that the establishment (or otherwise) of CM syndromes of hypercholesterolemia has direct relevance to the formulation of CHMs; CHMs are generally formulated to address the specific CM syndrome of a disease/disorder. There are particular categories of herbs that will be chosen to specifically address particular CM syndromes and particular aspects of pathogenesis of a condition. For example, if a diagnosis of “Spleen Qi Deficiency” is made, then herbs that “tonify” or strengthen the “Spleen” will be chosen from the Materia Medica. If a diagnosis is made of “Liver Yin deficiency” then quite different herbs will be chosen to address this. If there is “blood stagnation”, then herbs from the category of herbs that promote blood circulation will be chosen. Chinese medicinal formulae are created according to particular rules that are logical and structured within the framework of CM. 

## 5. Scientific Evidence about Chinese Herbs that Lower Cholesterol

Despite the uncertainty with respect to whether or not or which CM syndromes of hypercholesterolemia exist, what is known is that several Chinese herbs have been shown to have positive effects on lowering cholesterol. The list is extensive and includes the following: Dan Shen (Radix Salvia Miltiorrhiza), San Qi (Panax Notoginseng), Shan Zha (Fructus Crataegi), He Shou Wu (Polygonum Multiflorum [Polygonaceae]), Huang Lian (Coptis Chinensis), Da Suan (Bulbus Alli Sativi, garlic), Bai Guo (Ginkgo Biloba), Dang Gui (Angelicae Sinensis), Sheng Jiang (Rhizoma Zingiber Officinalis Recens, fresh ginger rhizome), Ge Gen (Radix Puerariae), Jue Ming Zi (Semen Cassiae), Gou Qi Zi (Lycium Barbarum), Pu Huang (Pollen Typhae), Du Zhong (Cortex Eucmmiae Oppositae), Shan Yao (Radix Dioscoreae Oppositae), Fu Ling (Sclerotium Poriae Cocos), Bai Zhu (Rhizoma Atractylodis Macrocephalae), Gua Lou (Fructus Trichosanthis Kirlowii), Gou Qi Zi (Fructus Lycii Chinensis), Sheng Di Huang (Radix Rehmanniae Glutinosae), Sang Ji Sheng (Ramulus Loranthi Seu Visci, Nu Zhen Zi (Fructus Ligustri Lucidi), Ju Hua (Flos Chrysanthemi Morifolii), and Ze Xie (Rhizoma Alismatis) [12, 17, 18]. 

Berberine, an alkaloid originally isolated from the Chinese herb Huang Lian (Coptis Chinensis), has been found to lower cholesterol in hypercholesterolemic patients and up-regulate hepatic LDL receptor expression [19]. It also has antihypertensive and antiarrhythmic actions [20]. Jue Ming Zi (Semen Cassiae) has been found to significantly increase HDL levels in hypercholesterolemic mice models compared with placebo [21]. In addition, Jue Ming Zi has been found to lower blood pressure [17]. Research has demonstrated that Pu Huang (Pollen Typhae) lowers serum cholesterol [18]. Ge Gen (Radix Puerariae) has been found to increase HDL cholesterol in patients with coronary heart disease [22]. Animal research has demonstrated that treatment of hypercholesterolemic rats with Pu Huang powder over six weeks significantly reduced serum cholesterol compared with placebo [23]. Extract of Ming Dang Shen has also been found found to significantly reduce serum cholesterol in rats [24]. Extract of Gou Qi Zi (Lycium Barbarum) has been found to have hypolipidemic, hypoglycaemic, and antioxidant effects in animal models; the antioxidant effect and hyperlipidemic effect were stronger in the water decoction and crude extracts compared with the polysaccharide fractions preparation whereas the opposite was true of the hypoglycaemic effect [25]. Bai Guo (Gingko Biloba) was found to positively affect reperfusion-induced ventricular arrhythmia in animals (though not the duration of the arrhythmia) and decreased the ischemia reperfusion-induced increase in CPK compared with controls [26]. 

As reported by Valli and Giardina [18], several studies have found that garlic (Chinese Da Suan) reduces serum cholesterol between 5% and 15% [27–30]. However, Berthold and Sudhop [31] caution that there is less convincing evidence of the effect of garlic preparations in lowering lipids on the basis of rigorously designed studies. Other studies have not found such positive results of garlic: one randomised controlled trial failed to show a significant difference from placebo in lowering cholesterol [32], and a meta-analysis (of five studies) failed to show a significant difference in LDL and HDL cholesterol compared with placebo [29]. In vitro and animal studies support a cholesterol lowering effect of Da Suan [33] and an anti-atherosclerotic effect [34] that is independent of lipid levels [18, 35, 36]. Garlic has been found to have fibrinolytic and antiplatelet activity and antihypertensive activity [18]. 

Tea consumption has been associated with lower CVD risk, and several animal studies have demonstrated a cholesterol lowering effect of both green and black teas [37]. Maron and colleagues found that intake of a green tea extract enhanced with theaflavin significantly lowered LDL cholesterol and total cholesterol compared with baseline in patients with mild-moderate hypercholesterolemia [37]. Mechanisms of action of green tea likely include upregulation of LDL receptors in hepatic HPG2 cells [38]. 

Red yeast rice appears to show promise as a herbal alternative for cholesterol lowering. Journoud and Jones [39] provide a very good review. Red yeast rice is produced by fermentation of rice with a specific strain of red yeast called Monascus purpureus. It has been used in China for centuries to make rice wine and flavour food and used in traditional medicine to treat digestive disorders [39–41]. Red yeast rice has been shown to lower total cholesterol by 13–26%, LDL cholesterol by 21–33% and triglycerides by 13–34%, and increase HDL cholesterol by 8–14% [39, 41]. Animal studies provide evidence that red yeast rice may reduce atherogenesis and induce nitric oxide-mediated endothelial-dependent vasodilation [42, 43]. It was banned from the US dietary supplement market in 2001 by the US Food and Drug Administration because one of the active constituents, Monacolin K (present in red rice that has been fermented with Monascus purpureus Went.), is the same as lovostatin that inhibits HMG-CoA reductase [40]. Other constituents of red yeast rice including plant sterols including *β*-sitosterol, campesterol and saponin, isoflavones, selenium and zinc may contribute to the cholesterol lowering ability of red yeast rice [40, 44] though Journoud and Jones [39] assert that generally there is little information about secondary bioactive constituents. Other possible mechanisms by which it may act include improving circulatory cholesterol clearance and inhibiting dietary cholesterol absorption [42] though the weight of evidence favours its inhibition of HMG-CoA reductase as the major mechanism [39]. Other types of pigmented rice including red and black rice show promise as cardio-protective functional foods [39]. Animal studies (rabbits) have shown that red and black rice can significantly reduce atherosclerotic plaque formation, raise HDL cholesterol and apoprotein A-I levels as well as reduce liver reactive oxygen species and aortic malondialdehyde levels, and increase liver total antioxidant capacity and erythrocyte superoxide dismutase activity [45]. Active constituents of red rice have been found to have radical-scavenging properties [46]. 

In terms of properties (temperature and taste characteristics) of the herbs as understood according to CM pharmacology theory, several of these herbs that lower serum cholesterol have “blood invigorating” or circulation-promoting properties including Dan Shen (Radix Salvia Miltiorrhiza), San Qi (Panax Notoginseng), (Dang Gui Angelica Sinensis), and Pu Huang (Pollen Typhae). Other herbs have an effect on eliminating “dampness” or “phlegm” such as Bai Guo (Ginkgo Biloba) and Huang Lian (Coptis Chinensis). Readers will remember from previous sections of the paper that from the CM theory perspective, it is believed that “blood stagnation” and “phlegm” are involved in the pathogenesis of hypercholesterolemia. Other herbs have actions that, from the CM perspective, are consistent with an ability to address the pathology underlying hypercholesterolemia. For example, He Shou Wu (Polygonum Multiflorum [Polygonaceae]) is a herb that “nourishes blood” and “tonifies” the Kidney and Liver Yin. Jue Ming Zi (Semen Cassiae ) belongs to the category of “Herbs that Drain Fire” and may address a syndrome of “Excess of Liver Yang” that is due to an underlying Liver and Kidney Yin deficiency (it also has a blood pressure lowering effect). Da Suan (garlic) and Sheng Jiang (fresh ginger) are warm in terms of temperature characteristic (according to CM pharmacology) and may address a syndrome where there is “interior cold” [17]. 

From a western pharmacological perspective, many Chinese herbs (including those mentioned previously) have other cardiovascular effects including reduction of blood pressure, ionotropic effects, cardiotonic effects, and cardio-protective effects including positive effects on cardiac perfusion, reduction of infarct size as a result of myocardial ischaemia, antioxidant effects, and improvements in other cardiovascular parameters [9, 17, 18, 20, 47, 48]. However not all effects of Chinese herbs on the cardiovascular system are positive, and drug-herb interactions are known [9]. 

Scientific evidence in relation to cholesterol-lowering activity of particular herbs is useful to the CM practitioner. Those herbs identified as lowering cholesterol belong to a range of categories of herbs within the CM Materia Medica. Thus, if a CM syndrome diagnosis is made in a patient with hypercholesterolemia, a choice may be made of the appropriate herbs that address the CM syndrome identified from amongst the many herbs that lower cholesterol, consistent with CM theory. 

### 5.1. Scientific Research into CHM

The effects of various Chinese herbal medicinal formulae on cardiovascular risk factors have been assessed in numerous studies though much of the research is limited to Chinese language publications. Several animal studies have demonstrated the beneficial effects of various CHMs in lowering cholesterol [49–53]. Other studies have demonstrated beneficial effects of herbal formulae on mechanisms involved in the atherosclerotic process including prevention of platelet adhesion and aggregation [54], enhancement of nitric oxide synthetase mRNA expression and inhibition of Endothelin mRNA expression in the vascular wall of atherosclerotic rabbits [55], improvement of vascular endothelial function in humans [56] and reduction in the extent of the atherosclerotic lesion, and decrease in severity of intimal proliferation (the formula tested was Shexian Baohe Pill. The study also found a reduction in serum cholesterol and LDL cholesterol but not HDL cholesterol or triglycerides)[51].

Lin and colleagues [49] found that intra-abdominal treatment of hypercholesterolemic rats and quails with “Kangshou liquid” (containing herbs Jiao Gu Lan, Huang Qi, Bai Zhu, Ze Xie, Dan Shen) was associated with a significant reduction in serum cholesterol and triglycerides compared with placebo. From the Chinese pharmacology perspective, the herbs in this prescription are herbs that address Spleen qi deficiency (Jiao Gu Lan, Huang Qi, Bai Zhu) and dampness (Ze Xie, Bai Zhu) and blood stagnation (Dan Shen). Treatment of hypercholesterolemic quails with *Dan Ze Compound* (herbs Dan Shen, Ze Xie, Jue Ming Zi, Shan Zha, Gua Lou Pi) was associated with a significant increase in HDL cholesterol compared with placebo [57]. The herbs in this prescription address blood stagnation (Dan Shen, Shan Zha), dampness (Ze Xie), Liver yang (Jue Ming Zi), and phlegm (Gua Lou Pi). The efficacy of these examples of formulae reported above lend some support to a pathomechanism involving dampness or phlegm and blood stagnation. On the other hand, it could also be argued that since the majority of these herbs individually have been shown to lower cholesterol using western scientific research methodology, the mechanism by which the herbal formulae work is entirely explained by western pharmacological evidence. The two types of evidence may support each other. 

Tu and colleagues [58] found that treatment of hyperlipidemic rats with a cardiovascular protective mixture “*CVPM*” (containing predominantly blood circulation-promoting herbs) significantly lowered total and LDL cholesterol and malondialdehyde levels, suggesting that CPVM is able to inhibit peroxidation of lipids compared with controls [58]. In addition, in vitro investigation of aorta samples of hyperlipidemic rats demonstrated that CVPM had protective effects on the vascular endothelium and that CVPM could also stimulate proliferation of endothelial cells [58]. Since according to CM theory this formula contains predominantly blood circulation-enhancing herbs, this particular study goes some way to extending the notion of “blood invigoration” beyond simply cardiotonic effects. CM theory should not stand still, simply resting on texts written two thousand years ago. Through exploring potential intersections of knowledge between CM and western medicine, scientific research has the potential to extend CM theory. 

Although not related to hypercholesterolemia, another interesting piece of research that examines intersections of knowledge is a study that investigated the relationship between CM syndromes related to “blood” and levels of platelet activation molecules [59]. Li and colleagues found that levels of platelet-activation molecules CD62P and CD63 were significantly raised in psoriatic patients compared with controls [59]. They subcategorised psoriatic patients into three CM syndromes (“blood heat”, “blood dryness,” and “blood stasis”) and found that CD62P and CD63 levels were significantly higher in patients with “blood stasis” syndrome compared with “blood heat” syndrome (the order of correlation was: blood stasis syndrome > blood dryness syndrome > blood heat syndrome) [59]. These results link objective western biochemical evidence of platelet activation (that plays an important role in higher blood viscosity, endothelial cell injury, and abnormal microcirculation) and the CM theory notion of “blood stagnation” as evidenced by clinical patterns of signs and symptoms. What is interesting from a CM perspective is that both “blood dryness” and “blood heat” may be earlier stages in the pathogenesis of “blood stagnation” (though they are not the sole pathways by which blood stagnation may occur and “heat in the blood” may not always develop into blood stasis, according to CM theory). 

## 6. Conclusion

Treatment of heart disease has a long history in China. The cardiovascular system in CM has several components of which the Heart is central, however differences can be seen between the traditional concept of the Heart in CM and the biomedical one. The Heart has an interdependent relationship with the other zang-fu organs. CM theory and pattern differentiation are now being applied to biomedically defined clinical entities such as hypercholesterolemia. CM theory has been applied to conceptualise the aetiology and pathogenesis of hypercholesterolemia, which includes the notions of phlegm and blood stagnation. There is a potential problem with this: hypercholesterolemia has no historical precedent of treatment prior to the rise of modern medicine. The appropriateness of applying pattern differentiation to hypercholesterolemia has not been established, despite the claims of authoritative texts. More investigations are needed with respect to this. 

With the imperative to become evidence based, CM is now being scrutinised in ways that it has not been previously. At a more basic level, modern science has demonstrated that a variety of individual Chinese herbs have cholesterol-lowering properties, and there are examples of clinical studies that have assessed the efficacy of medicinal formulae in lowering raised cholesterol. At a more sophisticated level, some researchers are challenging some of the very assumptions upon which CM is based and seeking intersections of knowledge between CM and biomedicine that may extend CM theory. In relation to hypercholesterolemia and CVD more generally, there is still much to explore. 
